# Management of Acute Posterior Multifocal Placoid Pigment Epitheliopathy (APMPPE): Insights from Multimodal Imaging with OCTA

**DOI:** 10.1155/2020/7049168

**Published:** 2020-03-13

**Authors:** Mariana A. Oliveira, Jorge Simão, Amélia Martins, Cláudia Farinha

**Affiliations:** ^1^Department of Ophthalmology, Centro Hospitalar e Universitário de Coimbra (CHUC), Coimbra, Portugal; ^2^Coimbra Institute for Clinical and Biomedical Research, Faculty of Medicine, University of Coimbra (iCBR-FMUC), Coimbra, Portugal; ^3^Association for Innovation and Biomedical Research on Light and Imaging (AIBILI), Coimbra, Portugal

## Abstract

A 28-year-old man presented to the emergency room with blurred vision in the right eye for two days. He reported a preceding flu-like illness one week earlier. His best-corrected visual acuity (BCVA) was 20/40 in the right eye and 20/25 in the left eye. There was no anterior chamber inflammation or vitritis in either eye. He presented multiple yellowish-white placoid lesions in the posterior pole, some involving the foveal area, bilaterally. General examination and systemic investigation were unremarkable. Multimodal evaluation with fluorescein angiography, indocyanine green angiography, and spectral domain and optical coherence tomography angiography (OCTA) were consistent with the diagnosis of acute posterior multifocal placoid pigment epitheliopathy. Due to centromacular involvement with decreased BCVA, treatment with oral methylprednisolone was started after infectious causes were ruled out. After two weeks, the patient presented functional and anatomical improvement. OCTA showed partial reperfusion of the choriocapillaris in the affected areas, in both eyes.

## 1. Introduction

Chorioretinal diseases may be a diagnostic challenge, and multimodal imaging is a strong resource in the management of these entities. This report describes the clinical course of a man with acute posterior multifocal placoid pigment epitheliopathy (APMPPE), documented by a multimodal approach, and especially with the recent introduction of OCTA in the clinical practice.

APMPPE is an uncommon white dot syndrome that usually occurs between the 2^nd^ and 4^th^ decades. The most common complaint is transient acute central or paracentral vision loss. First described by Gass in 1968 [1], it affects man and woman equally and is characterized by multiple whitish-yellow inflammatory lesions at the outer retina, retinal pigment epithelium, and choroid [2]. The etiopathogenetic mechanism of this entity is still not entirely clarified; however, it seems that the primary insult occurs at the level of inner choroid/choriocapillaris with retinal changes occurring secondarily [3, 4]. Some authors even discuss the current name of this entity, advocating for a change to choroidopathy rather than epitheliopathy [5].

We report a case of APMPPE with centromacular involvement, treated with systemic corticotherapy, with ocular fundus lesions and choriocapillaris reperfusion in optical coherence tomography angiography (OCTA), thus corroborating the predominant role of hypoperfusion of choriocapillaris in the etiopathogenesis of this disease.

## 2. Case Presentation

**Figure 1:**
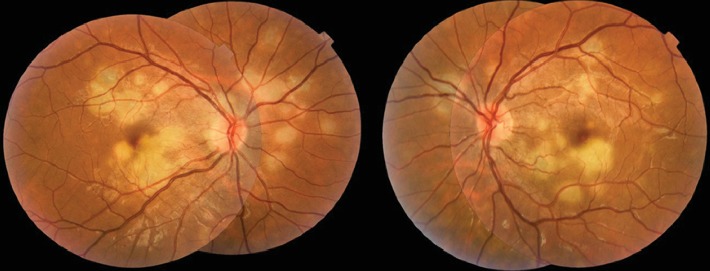
Bilateral presentation of fundus examination. Color fundus photography images show multiple bilateral yellow-white placoid lesions, at the level of external retina, posterior to the equator, and a slight fade of the edges of the optic nerve.

A fit-and-well 28-year-old man presented to our emergency room with a 2-day history of blurred vision in his right eye. He mentioned he always had lower VA in his left eye, and no prior history of ocular disease was reported. He had presented with myalgia and headache one week earlier, which resolved spontaneously. His BCVA was 20/40 in the right eye and 20/25 in the left eye. Slit-lamp biomicroscopy was unremarkable. Dilated fundus examination exhibited a hyperemic optic disc, peripapillary edema, and multiple yellowish-white placoid lesions in the posterior pole, some involving the foveal area, bilaterally (Figure 1). General examination was unremarkable.

**Figure 2:**
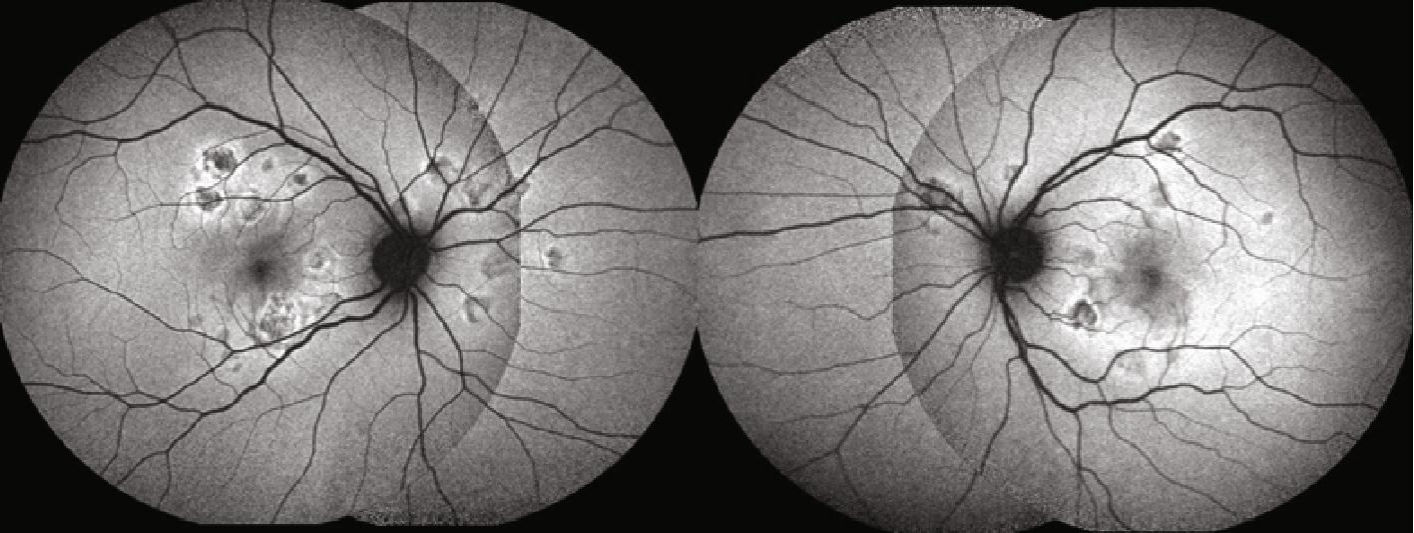
Bilateral fundus autofluorescence. Images show hypoautoflourescence corresponding to the placoid lesions, with a hyperautofluorescent edge.

**Figure 3:**
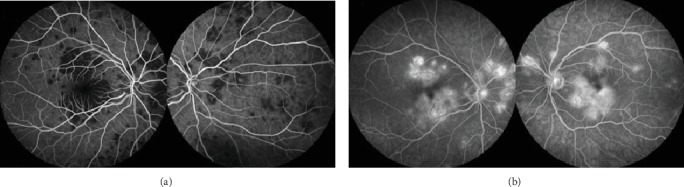
Representative fluorescein angiography (FA) images. FA typically showing early hypofluorescent placoid lesions (a) that become hyperfluorescent in the mid and late phases of the angiogram (b).

**Figure 4:**
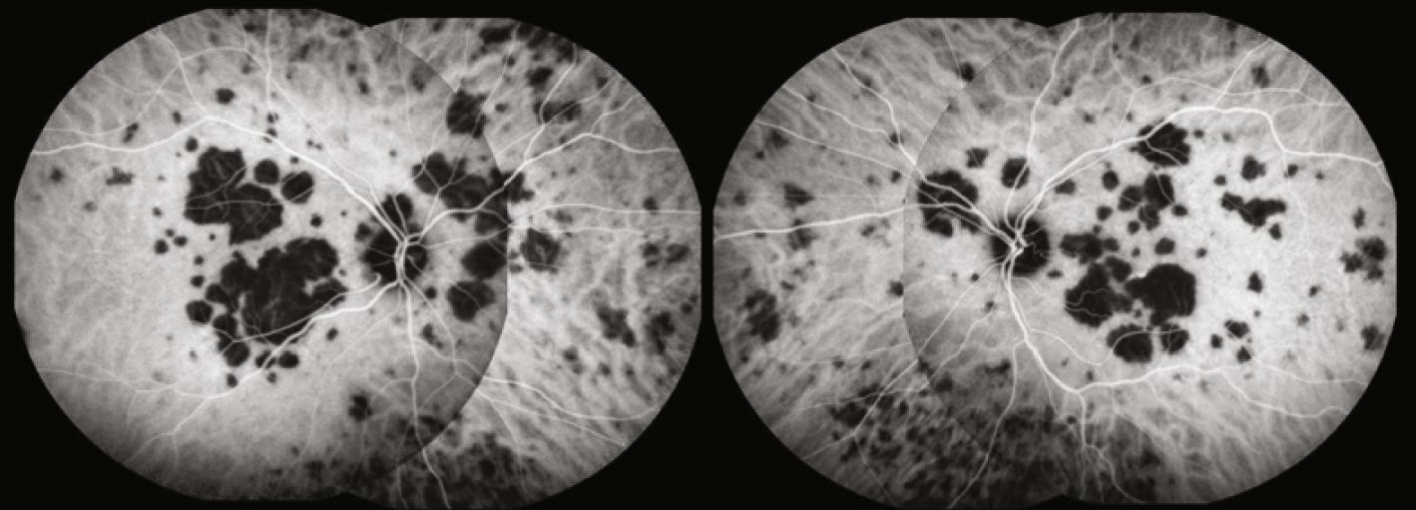
Representative indocyanine green angiography (ICGA) images. ICGA evidences hypofluorescence through the entire angiogram, corresponding to the placoid lesions.

**Figure 5:**
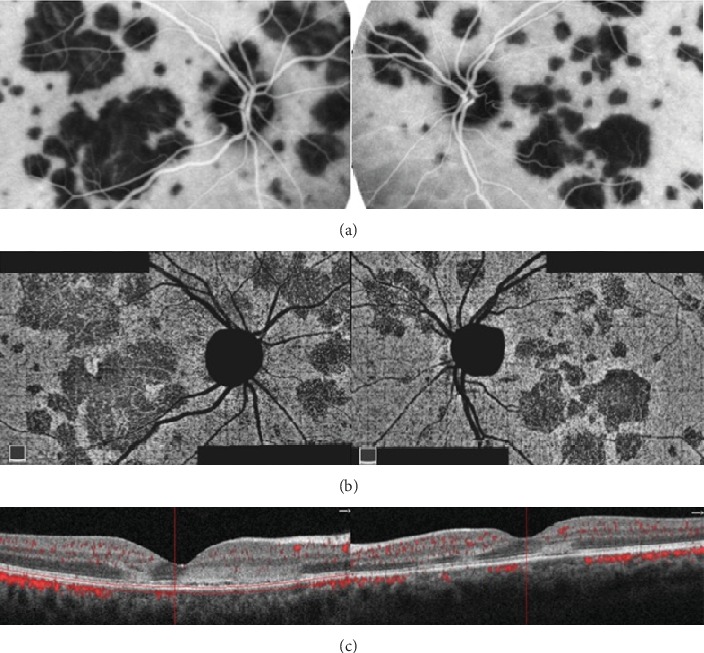
Multimodal evaluation of placoid lesions. (a) Indocyanine green angiography (ICGA) shows hypofluorescence corresponding to the placoid lesions; (b) optical coherence tomography (OCT) angiography demonstrates choriocapillaris ischemia in the corresponding lesions seen on ICGA; (c) OCT exhibiting hyperreflectivity from the outer plexiform layer to the RPE with normal retinal thickness.

Fundus autofluorescence (FAF) showed hypoautofluorescent lesions with an area of relative hyperautofluorescence along their edge (Figure 2). Hypofluorescence at the placoid lesions in the early phase that changed to hyperfluorescence at the late phases was evident on fluorescein angiography (FA) (Figures 3(a) and 3(b)). Indocyanine green angiography (ICGA) evidenced hypofluorescence of placoid lesions from early to late phases (Figures 4 and 5(a)). OCT (Avanti RTVue-XR 100, Optovue Inc., Fremont, CA) demonstrated bilateral focal areas of disruption of the ellipsoid layer and hyperreflectivity in the outer retina, primarily localizing to the outer nuclear layer (ONL) but also being seen at the level of the outer plexiform layer (Figure 5(c)). These features were more evident in the right eye. OCTA revealed significant hypoperfusion at the level of the choriocapillaris in the active lesions bilaterally (Figure 5(b)).

Due to the potential association with central nervous system (CNS) abnormalities, the patient was observed by the Department of Neurology. Neurologic examination, cranioencephalic-computerized tomography, and cervical and transcranial Doppler ultrasonography were unremarkable.

Extensive work-up to rule out other pathologies that could mimic APMPPE, including infectious diseases, was performed. This included serology for syphilis, herpes simplex 1 and 2, cytomegalovirus, Epstein-Barr, varicella zoster, Borrelia, HIV 1 and 2, and QuantiFERON-TB Gold test, which were all negative for active infection. Complete blood count, angiotensin conversion enzyme, hepatic enzymes, renal function, and thorax X-ray were unremarkable. C-reactive protein and sedimentation velocity were increased (3.86 mg/dL and 31 mm/h, respectively).

Regarding the differential diagnosis, we excluded syphilis, tuberculosis, other infectious causes, Vogt–Koyanagi–Harada disease, and sarcoidosis, based on the history, the results of laboratory tests, and the lack of systemic findings. The presence of a previous viral prodrome, the young age, and the multiple yellowish-white placoid lesions in the posterior pole pointed to an inflammatory choriocapillaropathy. The morphology of the lesions helped in the discrimination of the white dot syndrome, as the placoid conformation with lesions bigger than 125 *μ*m with a low degree of inflammation was in favor of APMPPE or serpiginous choroiditis. However, the choroiditis' geographical pattern with centrifugal extension from the peripapillary region typical of serpiginous choroiditis was not observed [6]. Instead, the deep multiple yellowish-white placoid lesions in the posterior pole associated with a minimal vitreous inflammation in a young healthy male with a negative QuantiFERON-TB Gold test are favorable of the diagnosis of APMPPE. The fact that the lesions resolved over time without recurrences was also in favor of this diagnosis. There are relentless serpiginous and ampiginous forms that can mimic APMPPE.

The placoid lesions in APMPPE spontaneously resolve in most cases, and VA usually recovers within a few months [7]. However, there are reports of permanent changes and therapy with corticosteroids may have a role when there is foveal involvement, advanced age, unilateral disease, and recurrences or risk of central nervous system vasculitis [8]. Due to centromacular involvement, the patient started treatment with 1 mg/kg/day of methylprednisolone.

**Figure 6:**
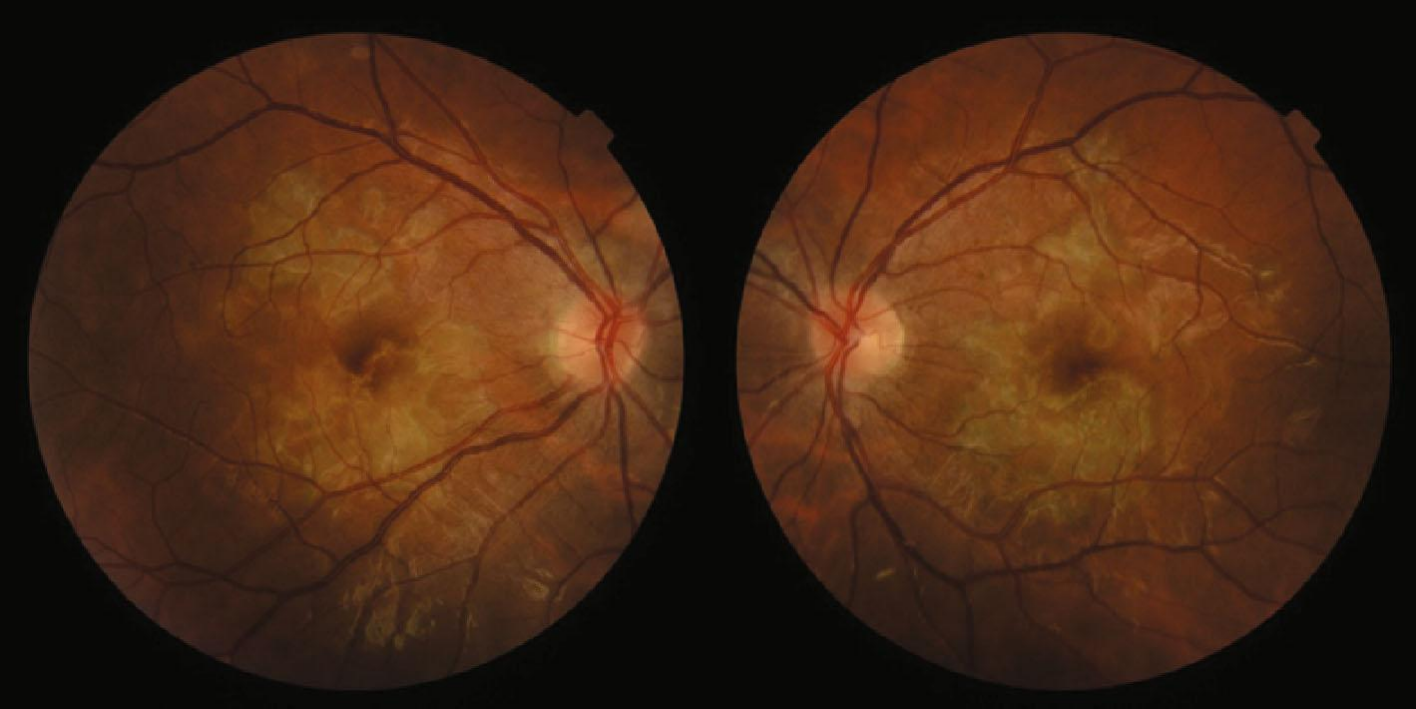
Bilateral presentation of fundus examination, after two weeks of follow-up. Color fundus photography images show the placoid lesions in resolution.

**Figure 7:**
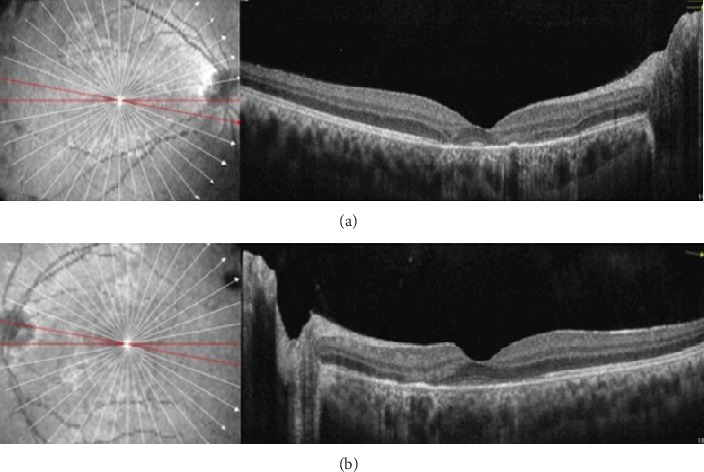
Optical coherence tomography of the right (a) and left (b) eyes after two weeks of follow-up. OCT demonstrated resolution of macular edema and focal atrophy of outer retinal layers, from the outer plexiform layer to the retinal pigmented epithelium, corresponding to the previous hyperreflective areas.

**Figure 8:**
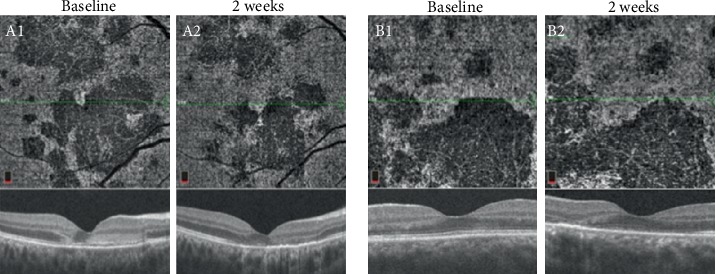
Optical coherence tomography angiography (OCTA) images at the level of choriocapillaris revealing focal areas of hypoperfusion in the right (A1 and A2) and left (B1 and B2) eyes. After two weeks, the area of hypoperfusion at the level of the choriocapillaris improved and the thinning of the outer retina in spectral domain optical coherence tomography persisted.

After two weeks, the patient presented functional and anatomical improvement. BCVA was 20/30 in his right eye and 20/22 in the left eye. Slit-lamp examination remained unremarkable, and at dilated fundus examination, it was possible to note placoid macular lesions in resolution (Figure 6). No *de novo* lesions were observed. SD-OCT demonstrated resolution of macular edema and focal atrophy of outer retinal layers corresponding to the previous hyperreflective areas (Figure 7). OCTA showed reperfusion of the choriocapillaris in the affected placoid areas, in both eyes (Figure 8).

At this time, methylprednisolone was tapered off to 48 mg/day and a reevaluation was scheduled to two weeks later. Unfortunately, the patient missed these and the subsequent appointments. A head magnetic resonance imaging requested by the neurology department was also missed.

## 3. Discussion

Acute posterior multifocal placoid pigment epitheliopathy is a nongranulomatous chorioretinitis of uncertain origin that occurs in healthy young adults. Frequently, as we observed in our patient, a viral prodrome precedes the onset of ophthalmologic symptoms [1, 7]. This fact raises the question of an infectious agent as the trigger for the illness [9–11].

Although the ophthalmic findings may reflect RPE involvement, accumulating evidence indicates that the primary lesion is posterior to this and choroidal perfusion is abnormal. Secondary ischemic changes would produce disruption of the pigment epithelium, resulting in typical placoid lesions [12, 13]. Nowadays, OCTA allows visualization of the rarefaction of the choriocapillary at corresponding placoid lesions seen in the fundus at the acute stage of the disease [14].

There is a picture developed that APMPPE is an acute, autolimited monophasic inflammatory illness with a typical clinical pattern, sometimes with profound loss of vision, but usually with remarkable visual recovery despite substantial residual scarring of the RPE [1, 15]. Though subtotal recovery of VA is usual, many patients have long-term visual symptoms and some have significant residual field defects [7]. Eyes without initial foveal involvement evidence final better functional prognosis (87.5% have a BCVA higher than 20/25 versus 39.2% when there is foveal involvement) [16, 17]. In fact, a report of 33 eyes with APMPPE observed that in the 7 eyes in which VA failed to recover to better than 20/80, all had foveal involvement. They also presented one of the following atypical features: age older than 60 years, unilaterality, an interval before involvement of the second eye of at least 6 months, and recurrence of the disease [18]. In rare cases, typical signs are also associated with progressive deterioration, with widespread severe choroidal atrophy after apparent clinical healing of the placoid lesions [19].

In this case, we did not observe uncharacteristic features, although foveal involvement was initially present. For this reason, we initiated therapy with oral corticosteroid. Data from literature does not provide clear information regarding the use of oral corticosteroids. Cases describing the use of steroids advocate it is possible that it modifies the natural course of lesions, although the role of this treatment in the eventual resolution of this clinical condition remains speculative, that is, the choroidal reperfusion may also occur spontaneously in the course of the disease, without steroids [12, 16, 20]. When there is central nervous system involvement, it is stated that intravenous corticosteroids associated with immunosuppressive therapy should be considered. In a case series of patients with APMPPE and SNC lesions, all patients were submitted to treatment with corticosteroids and in some of them, VA decreased when they were tapered off [8].

A recent publication distinguished 4 phases of APMPPE based on multimodal imaging: choroidal, chorioretinal, transitional, and resolution [4]. Lesions in phase 1 (choroidal) can resolve without any morphological sequelae. Our patient presented in phase 2, as there was evidence of classic active lesion on FA with early hypofluorescence and late hyperfluorescence, persistent hypocyanescence of lesions across ICGA frames, and choroidal hypoperfusion on OCTA. There was also loss of structural integrity and hyperreflectivity of the outer retinal layers including RPE, ellipsoid layer, and ELM on SD-OCT and predominant hypoautofluorescent of APMPPE lesions noted on FAF.

As we observed with OCTA, the reperfusion of choriocapillaris was only partial and can occur in a centripetally pattern from the outer edge of the APMPPE lesion. This was also previously described [4]. In the follow-up visit, persistent thinning of the outer retina was visible on SD-OCT with hyporeflectivity at the level of RPE, which was consistent with phase 4 in these areas.

Multimodal imaging provides valuable information about the clinical course of APMPPE. Despite the utility of FA and ICGA images for monitoring disease activity and the very typical pattern, these are invasive techniques, and due to the blocking effects, they do not allow to locate the depth of the primary lesion or to characterize it. On the other hand, OCTA enhances understanding of the hypoperfusion choriocapillaris involvement of lesions, noninvasively. In our case, we could identify previously described progression patterns of this entity and document good response to treatment by a noninvasive method.
